# Indigenous-led struggles for health justice in the context of the climate emergency: insights from Guatemala

**DOI:** 10.1136/bmjgh-2024-015519

**Published:** 2024-11-28

**Authors:** Jeannie Samuel, Benilda Batzin, Rosaura Medina, Evaristo Caal, Karin Slowing, Esteban Sabbatasso, Walter Flores

**Affiliations:** 1 Health & Society, York University, Toronto, Ontario, Canada; 2 Dahdaleh Institute for Global Health Research, York University, Toronto, Ontario, Canada; 3 CERLAC, York University, Toronto, Ontario, Canada; 4 Centro de Estudios para la Equidad y Gobernanza en los Sistemas de Salud (CEGSS), Guatemala City, Guatemala; 5 Laboratorio de Datos GT, Guatemala City, Guatemala; 6 Politics, York University, Toronto, Ontario, Canada; 7 Accountability Research Center, School of International Service, American University, Washington, DC, USA

**Keywords:** Global Health, Environmental health, Health education and promotion, Health policy, Public Health

## Abstract

This practice paper reflects on an ongoing Participatory Action Research project that combines community-engaged methods, national data analysis and advocacy to support community-based emergency response to extreme weather events in 16 Indigenous communities in Alta Verapaz province, Guatemala. Our work points to a worrying predicament experienced in climate-affected areas, where some populations face a dangerous confluence of climate vulnerability, social exclusion and state abandonment that imperils human health. Indigenous communities in Alta Verapaz are often particularly vulnerable to health impacts from climate-driven extreme weather events, a reality compounded by the historical and contemporary ways the state marginalises them. We share work from our project activities to shed light on these interconnected problems and how Indigenous communities in Alta Verapaz, especially Maya Q’eqchi’ communities, are using creative strategies to confront them. Technical solutions are important but insufficient responses. Community-led activism to push for state support to address extreme weather events, as has been practised in struggles for health rights, can provide vital tools for addressing the increasing challenges these populations face in the context of the climate crisis.

**SUMMARY BOX**

- Many Indigenous communities are becoming increasingly exposed to health impacts from extreme weather events.
- This reality is compounded by the interconnected historical and contemporary ways the state marginalises these Indigenous communities.
- Indigenous communities in Alta Verapaz, Guatemala, receive minimal emergency response services from the state despite repeated extreme weather crises.
- Community-led activism to push for increased response from the state to address extreme weather events can provide vital tools for addressing the increasing challenges these communities face in the context of the climate crisis.
- This Participatory Action Research project facilitates the understanding of complex realities faced by Indigenous communities affected by climate change. It also supports strategies to demand improved state response at different levels of government.

## Introduction

 According to the European Union’s Copernicus Climate Change Service, 2023 was the hottest year on record, with global temperatures already 1.48°C higher than the preindustrial average.^1^ Climate change is fueling extreme weather events that threaten human health and well-being in diverse ways including through extreme heat, wildfire smoke, contaminated flood water, as well as loss of access to shelter, food, infrastructure and livelihoods.^2^ For populations impacted around the world, climate change is not a future event but rather a reality being lived today. This is particularly true for disadvantaged groups living in regions that are now experiencing increasingly frequent emergencies.^3^ This practice paper reflects on an ongoing Participatory Action Research (PAR) project that combines community-engaged methods, national data analysis and multilevel advocacy to support community-based emergency response to extreme weather events in 16 Indigenous communities in Alta Verapaz province, Guatemala.

We are an interdisciplinary team composed of Indigenous researcher/practitioners and allied members. As coauthors of this article, we are from diverse social and professional locations, including three Mayan researcher/practitioners who work with the Guatemalan NGO, Centro de Estudios para la Equidad y Gobernanza en los Sistemas de Salud (CEGSS), two of whom are Maya Q’eqchi’ from Alta Verapaz, the area of focus in this article. We also have four allied team members: a national policy analyst from the Guatemalan NGO, Laboratorio de Datos GT, and three researchers based at universities in Toronto, Canada and Washington DC, USA. Together with rural Indigenous communities, we are implementing a 3-year PAR project. We understand PAR as ‘a scholar–activist research approach that brings together community members, activists and scholars to cocreate knowledge and social change in tandem’.^4^ PAR is typically centred on collaboration between communities with lived experience and knowledge of a social issue and professional researchers with other relevant skills, expertise and resources. The research relationship aims to create knowledge, not for its own sake, but in service of partner communities and their goals.^4^ Our project is the result of demands from communities in Alta Verapaz for accompaniment from civil society organisations they trust, CEGSS and Red de Defensores y Defensoras Comunitarios por el Derecho a la Salud (REDC-Salud), to understand why government agencies are not helping them to mitigate the effects of climate change, including extreme weather events. The project aims to create strategies to demand government’s attention to address these problems.

## Context: Alta Verapaz, Guatemala

**Figure 1:**
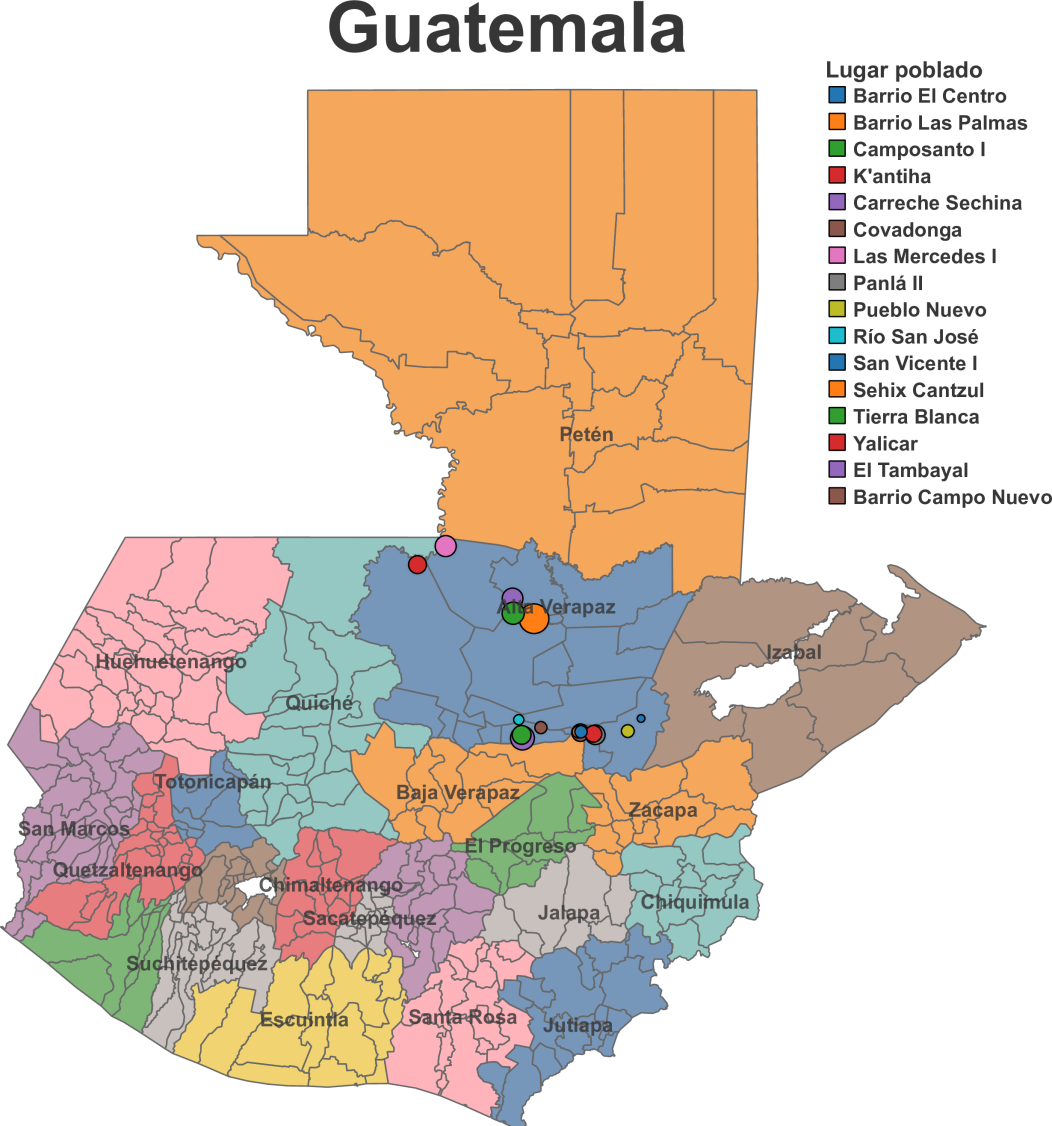
Map of project locations in Alta Verapaz, Guatemala. (Source: Laboratorio de Datos GT, 2024).

Our current work takes place in 16 communities in 5 municipalities in Guatemala’s mountainous department of Alta Verapaz (figure 1). Guatemala is a country in Central America with a population of approximately 17.3 million. Despite economic growth, the country’s poverty and inequality rates are among the highest in Latin America and the Caribbean.^5^ For many years, the state of Guatemala has been characterised by low taxes and spending, and publicly provided basic services in the country have been chronically weak and under-resourced.^5^

According to census data, 43.75% of Guatemala’s population is Indigenous, including Maya, Xinka and Garifuna peoples. The largest Indigenous group is the Mayan people, a broad ethnic category comprising 22 distinct groups with diverse languages and cultures.^6^ Alta Verapaz is a predominantly Indigenous area with 92.95% of its population identifying as Maya, mostly belonging to the Q’eqchi’ people.^6^ Alta Verapaz is one of Guatemala’s most marginalised departments in the country, as reflected in its socioeconomic and health indicators. According to recent statistics, 83% of the population in Alta Verapaz lives in poverty.^7^ Contemporary government services in the department suffer from a lack of state concern and investment, compounded by community mistrust—itself a legacy of colonialism and the ongoing racism and discrimination directed at Indigenous peoples.^8^

The present situation in Alta Verapaz, particularly the struggle over land, is also marked by Guatemala’s troubled past, commencing with the highland region’s history as a site of enduring resistance to colonial rule in the 1500s. By the 1870s, Indigenous communities in Alta Verapaz faced accelerating encroachment when state rules favoured the introduction and expansion of coffee plantations by German settlers. Indigenous peasants in Alta Verapaz were dispossessed to such a degree that by the 1930s the great majority became dependent agricultural labourers within the plantation system.^9^ From 1960 to 1996, Guatemala suffered a civil war in which more than 200 000 were killed and Indigenous populations throughout the highlands were displaced.^10^ Today, in the postconflict period, Indigenous communities in Alta Verapaz face incursions from industrial megaprojects, including mining, dams and agroindustrial plantations, that many view as ongoing threats to their lands and culture.

Alta Verapaz is an area of the country where storms, floods and landslides are becoming common occurrences, with grave results.^3^ For example, pre-existing precarity in the department of Alta Verapaz left the population especially vulnerable when Tropical Storms Eta and Iota hit in 2020. A disproportionate number of the 311 000 people forced to evacuate their homes were concentrated in Alta Verapaz. The department also suffered the worst damage and losses to their health facilities from the storms.^11^ The World Bank notes that extreme weather events and other disasters have damaged infrastructure, harmed agricultural output, exacerbated food insecurity, spread diseases and disrupted essential services.^5^ With weak state emergency response to these extreme weather crises, social cohesion and community resilience are more important than ever.

Our work in Alta Verapaz points to a worrying predicament experienced in climate-affected areas, where some populations face a dangerous confluence of climate vulnerability, social exclusion and state abandonment that imperils human health. In this practice paper, we share insights from project activities in Alta Verapaz to shed light on these interconnected problems and how they are being confronted. The understanding we are gaining through our PAR in Guatemala suggests that technical solutions are important but insufficient responses to the complex dynamics in Alta Verapaz. Forms of community-led activism, as have been practised in struggles for health rights, may provide important tools for addressing the increasing challenges these populations face in the context of the climate crisis.

In the subsequent sections of our paper, we discuss the context and central problem our project addresses, then reflect on and analyse our project’s process to date, considering how what we are learning can be applied to settings beyond Guatemala. We briefly conclude by sharing the next steps.

## Protecting human rights in the climate crisis

The World Bank has ranked Guatemala as the ninth most at-risk country for negative ramifications from climate change.^12^ Extreme weather events have become more frequent and more dangerous, making rural populations, the majority of whom are Indigenous, particularly vulnerable. In recent years, many communities have found themselves in a state of chronic emergency, unable to fully recover from one extreme weather event before they are hit by another. This corresponds with an established global pattern where climate vulnerability and social exclusion are found to be related.^3^ Rural Indigenous populations, long marginalised in Guatemalan society, often occupy precarious and flood-prone lands due to historical displacement by European colonisers and the economic elite. In this difficult context, Indigenous communities frequently demonstrate innovative forms of collective action and solidarity in response to extreme weather events. However, members of these communities are disproportionately represented among the extreme poor, and many communities lack sufficient financial and technical resources to withstand repeated climate emergencies.

The Inter-American Commission on Human Rights (IACHR) recognises this relationship between marginalisation and climate vulnerability in its 2021 resolution on state human rights obligations related to the climate emergency.^13^ The Commission observes that populations face ‘differentiated impacts’ and unequal vulnerabilities to the effects of climate change. While states are of course responsible for protecting the rights of all, the resolution emphasises they also have a ‘reinforced obligation’ to protect vulnerable groups such as those burdened by structural inequality who may be particularly exposed to the impacts of climate change. The IACHR highlights Indigenous peoples, Afro-descendant, tribal or peasant communities, the rural poor, women and girls, migrant workers, as well as other groups facing intersecting forms of marginalisation.^13^ The IACHR resolution is echoed in resolutions by other global human rights bodies.^14^

## Indigenous communities and state abandonment in Guatemala

Given the IACHR and other global bodies’ particular emphasis on protecting the rights of vulnerable groups in the context of the climate crisis, it is striking that in Alta Verapaz the impacts of the climate emergency are compounded by the state’s failure to provide effective emergency response to support communities hit by extreme weather events, especially floods and landslides. On paper, Guatemala has a robust and well-thought-out emergency response system. Its risk management and humanitarian assistance system is governed by a specific law on Risk Management and Disaster Assistance. There is a dedicated institutional framework and a mandate for articulation between all government ministries and secretariats. As well, there is a recently updated national disaster response policy and plan, plus several other relevant instruments and resources. The system should allow for an effective and timely response to the situations experienced by the population.^15^ In practice, however, this system barely exists for communities in Alta Verapaz. Important parts of the system are neglected. In 2023, communities involved in our project informed us that humanitarian aid that should flow to their communities was sometimes diverted to government supporters, whether at the municipal, departmental or national level. Some community partners also tell us that being designated as a high risk area means they can be excluded from government investment plans, but this designation has not enabled them to receive emergency assistance.

We understand this gap between formal state policy and grounded reality for Indigenous communities in Alta Verapaz as a form of what the geographer Ruth Gilmore and others conceptualise as ‘organised abandonment’.^16^ This refers to how marginalised populations can be devalued and made vulnerable as a result of the neglect or inappropriate action of the state and other organisations. It is one of several terms used to describe the kinds of structural oppression experienced by socially excluded populations, particularly those enduring a legacy of colonialism and/or racialised exploitation. It points to how some groups are in effect deemed unworthy of safety and security, and to how their abandonment can create economic opportunities for more powerful actors.^17^ In Guatemala, Indigenous people have a long history of marginalisation and oppression, from the colonial era, through the civil war (1960–1996), to the current postconflict period. In regions like Alta Verapaz, with a history of struggles over land, ineffective emergency response also serves certain political and economic interests since the dislocation of Indigenous communities can weaken opposition to the expansion of resource extraction and palm plantation projects favoured by national elites. This is an example of what Rivera calls ‘disaster colonialism’, referring to the role played by disasters and disaster response in perpetuating colonial dispossession.^18^

In other words, Indigenous communities in Alta Verapaz are often particularly vulnerable to health-related climate impacts, a reality compounded by the interconnected historical and contemporary ways the state marginalises them. Through this process, the state also fails to fulfil several of its human rights obligations, including obligations that promote health, as outlined in the IAHRC’s Resolution 3/21 in the context of the climate emergency.

## Partnering with Indigenous front-line health defenders and climate-affected communities

In Guatemala, we are learning more about these complex dynamics through our partnership with RedC-Salud, a national network of community-based Indigenous health rights activists. Known as Frontline Health Defenders (FHDs), these activists have been struggling with the state to decolonise the publicly provided health services available to their communities. For the past 10 years, RedC-Salud members have developed multilevel advocacy strategies to promote accountability and combat racism, cultural insensitivity and poor service in rural healthcare.

In this project, we are working in collaboration with affected communities in Alta Verapaz and in partnership with the FHDs, in their capacity both as advocates and community researchers. Over the past year, we have engaged in participatory mapping in 16 communities about the problems they face during recurring extreme weather events, and to identify the emergency response activities carried out by the state, other actors and the communities themselves. These mapping sessions were conducted in Q’eqchi, the main Indigenous language in the area, and cofacilitated by two Indigenous practitioners from one of the partner NGOs (CEGSS) together with Q’eqchi’ FHDs from Alta Verapaz, members of REDC-Salud.

During the mapping sessions, community members identified serious challenges related to extreme weather events. They noted immediate problems faced during emergencies, including a lack of safe evacuation routes and transport, and a lack of emergency shelter accessible to all affected community members. They also highlighted longer-term challenges. For example, repeated flooding during the rainy season contaminates their drinking water due to overflowing latrines, leading to health problems including diarrhoea, vomiting, headaches and skin rashes. Crucial reproductive health needs are also often unmet during these events. One woman reported having to give birth in a makeshift emergency shelter when her community was forced to evacuate during a set of storms. She never received any support or follow-up care from her local state health services. Focus group participants in Alta Verapaz reported extreme stress and negative repercussions for their mental health from constant worry in anticipation of the next extreme weather event. Flooding also ruins their crops and harms their livestock, resulting in severe hunger and increased food insecurity (May 2023). Despite the gravity of these events, these 16 Indigenous communities in Alta Verapaz reported receiving little or no government assistance during or following their times of crisis. Instead, communities largely managed their own disaster response and recovery, self-organising and helping one another the best they could. This is consistent with the national data analysis conducted by another of our Guatemalan project partners, Laboratorio de Datos.^15^

We noticed in Alta Verapaz that a few of our community partners already have existing community risk maps that they developed in the past with other NGOs and international cooperation. These previous maps provide important baseline information from which we can see the increasing impact of extreme weather events on community geography and infrastructure. However, the earlier maps focus narrowly on community resources and do not explicitly direct attention to the state as a potential source of support and service provision. In our view, community risk maps are an important interactive learning tool, but they are not an effective product on their own. Maps provide necessary information and analysis, but collective action is required to help translate this into change. Together with REDC-Salud, we are working to design advocacy strategies that FHDs and communities can use to push municipal and higher-level officials to recognise the Guatemalan state’s obligations to protect their rights, including health rights, during climate-driven emergencies.

**Figure 2:**
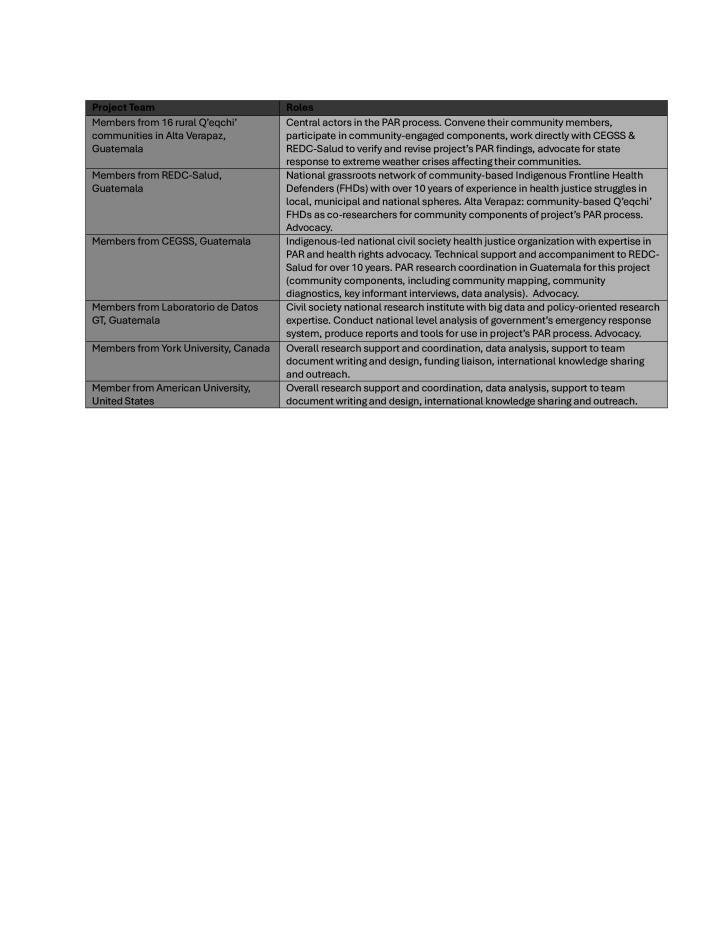
Summary of project members and roles. (Source: Building Community-Based Emergency Response Strategies Project Team, 2024).

In November 2023, a few months after our initial community mapping sessions, these lessons were sadly confirmed when several of the partner communities experienced serious flooding. Hit with life-threatening levels of water, at least two of the communities were forced to evacuate. No response from the state was immediately forthcoming. One of our NGO project partners and several of the FHDs used materials and information gleaned from the mapping process to press government officials for emergency support. They attracted media coverage and even individual private donations. But official state assistance barely materialised. This reaffirms our understanding of the gravity of the crisis and our analysis around state abandonment, its impacts on communities’ mental and physical health, and the need for our project to continue to work alongside FHDs and partner communities to build advocacy strategies for change (figure 2).

## Moving forward: FHDs and advocacy for emergency response

A summary of project members and roles is outlined in figure 2. In carrying out this work, two things have become apparent. First, as emergencies become more frequent, the FHDs in Alta Verapaz are expanding their roles to help lead the grassroots response to extreme weather events in their communities. While they may lack formal training in disaster management, the FHDs have intimate knowledge of, and strong personal connections to, places and communities in the affected area. The emergency management literature recognises that such actors are an important part of ‘community resilience’ for disaster risk reduction and can play a pivotal role in overall disaster response. This scholarship advises donors and public officials to build local capacity through training and developing ‘coalitions’ of actors and organisations able to coordinate local response with state authorities.^19 20^ However, in Guatemala at present, the state has had minimal direct involvement in such efforts. A recent change of national government may offer renewed potential to strengthen the state’s emergency response system in support of rural and Indigenous communities. However, it is too early to know.

Second, our experience suggests that FHDs may be especially well positioned to address the failure of the state’s emergency response framework. For example, over the past 10 years, the network of FHDs has built considerable experience engaging in struggles with the state in relation to healthcare services through coordinated advocacy efforts and engagement with officials. Currently, the FHD network, in collaboration with affected communities, is expanding its work to more Indigenous municipalities. In these circumstances, pressuring the state to step up not only on paper but in practice is likely to play a vital part in effective emergency planning and response. Given the myriad ways extreme weather events impact health, these advocacy efforts are part of wider struggles for health justice. The experiences of the FHDs (and research on grassroots health rights advocacy) suggest that, despite structural challenges, state service provision is a contested arena where advocacy can produce at least some degree of success.^21 22^ Advocates can use strategic opportunities to mobilise legal frameworks, local bureaucracies, human rights tools and discourses of citizenship and equal rights to press for accountability. More broadly, these efforts form part of larger political movements in favour of inclusive change and decolonising state institutions. They also serve to emphasise the inextricable links between climate change and health and the health costs that chronic extreme weather emergencies are having on communities.

In the year to come, project partners will continue to develop advocacy strategies and community-based emergency response plans that include dialogue with the state at municipal, province and national levels. They will also advocate for state-provided health services, urging them to better recognise and respond to their specific health needs arising from increasingly frequent extreme weather events.

The expansion of the work by FHDs is not without cost. It places even greater stress and pressure on an already overstretched segment of civil society already deeply engaged in health and human rights struggles on behalf of their communities. They report increased physical and mental health impacts on communities hit by extreme weather events, especially flooding. We were struck by the comment of one FHD in Alta Verapaz who claimed that, given the increasing number of floods in the context of climate-related extreme weather, and the slow response of the state to this new reality, half the year they need a boat more than an ambulance (April 2023). While certainly ambulances are still essential health system inputs, the comment illustrates how organised abandonment, in the context of a climate emergency, downloads responsibilities to the community level, including community-based health actors. We cannot disentangle the realities of political and economic weaknesses from its impacts on local communities, and in this case, FHDs and other community-based health actors compelled to deal with the current climate emergency.

## Conclusion

In closing, our discussion about the situation in Alta Verapaz, Guatemala suggests that populations in climate-affected areas can face a dangerous confluence of climate vulnerability, social exclusion and state abandonment that threatens health and other human rights and well-being. Technical responses alone are likely to have limited effects without political, rights-based struggles that use a decolonial lens to challenge the historically entrenched inequalities, including health inequalities, experienced by vulnerable groups. The climate crisis is multifaceted and requires profound transformations, particularly the shift away from fossil fuels,^23^ but also increased attention to marginalised groups at the most risk of harm. In responding to these challenges, we need to value and recognise innovative, community-engaged resistance practices as well as expertise from diverse worldviews. For example, in the case of FHDs and partner communities in Alta Verapaz, there is much we can learn from Indigenous Mayan worldviews, including specific Maya Q’eqchi’ worldviews, which emphasise a close relationship with territory as well as balance, harmony and full respect for other living beings. Equally, there is much to be learnt from the decolonising struggles and strategies used by Indigenous community-engaged activists and others from marginalised groups.

We urge expanded dialogue among scholars, practitioners, policy-makers and community-engaged advocates in the global health arena to better understand the complexities that drive the health impacts of the current climate crisis on socially excluded communities, as well as to learn from innovative response strategies in diverse settings.

## Data Availability

All data relevant to the study are included in the article or uploaded as online supplemental information.
